# Fruit and Vegetable Consumption and Cardiovascular Risk Factors in Older Chinese: The Guangzhou Biobank Cohort Study

**DOI:** 10.1371/journal.pone.0135380

**Published:** 2015-08-10

**Authors:** Yangbo Sun, Chao Qiang Jiang, Kar Keung Cheng, Wei Sen Zhang, Gabriel M. Leung, Tai Hing Lam, C. Mary Schooling

**Affiliations:** 1 School of Public Health, Li Ka Shing Faculty of Medicine, The University of Hong Kong, Hong Kong, China; 2 Guangzhou Number 12 Hospital, Guangzhou, China; 3 Department of Public Health and Epidemiology, University of Birmingham, Birmingham, United Kingdom; 4 School of Public Health, CUNY, New York, United States of America; University of Kentucky, UNITED STATES

## Abstract

**Objective:**

To examine the adjusted associations of fruit consumption and vegetable consumption with the Framingham score and its components in the non-Western setting of Southern China, considering health status.

**Method:**

Linear regression was used to assess the cross-sectional associations of fruit and vegetable consumption with the Framingham score and its components, among 19,518 older Chinese (≥50 years) from the Guangzhou Biobank Cohort Study in Southern China (2003–2006), and whether these differed by health status.

**Results:**

The association of fruit consumption with the Framingham score varied by health status (*P*-value<0.001), but not vegetable consumption (*P*-value 0.51). Fruit consumption was associated with a lower Framingham score (-0.04 per portions/day, 95% confidence interval (CI) -0.08 to -0.004) among participants in poor health, adjusted for age, sex, recruitment phase, socio-economic position and lifestyle. However, similarly adjusted, fruit consumption was associated with a higher Framingham score (0.05, 95% CI 0.02 to 0.09) among participants in good health, perhaps due to a positive association of fruit consumption with fasting glucose. Similarly adjusted, vegetable consumption was associated with a higher Framingham score (0.03, 95% CI 0.01 to 0.05) among all participants, with no difference by health status.

**Conclusion:**

This large study from a non-western setting found that fruit and vegetable consumption was barely associated with the Framingham score, or major CVD risk factors.

## Introduction

Fruits and vegetables are promoted as part of a healthy diet. For example, Dietary Guidelines for Americans (2010) suggest 2.5 cups of vegetables and 2 cups of fruit daily [1], while guidelines for Chinese recommend 4–6.5 portions of vegetables and 2.5–5 portions of fruit daily (80 grams/portion is a usual serving size) [2].Observationally in Western settings fruit and vegetable consumption is associated with lower risk of cardiovascular disease (CVD) [3,4], diabetes [5] and cancer [6]. Fruits and vegetables contain vitamins, fiber, minerals, including calcium and magnesium, and bioactive phyto-chemicals, including carotenoids and phenolic compounds [7], which may protect against CVD and its risk factors via antioxidants, or by fiber preventing excess nutrient uptake [8,9]. The benefits of vitamins and minerals have not been substantiated by randomized controlled trials (RCTs) [10,11], but RCTs indicate that the Mediterranean diet, rich in fruits and vegetables, fish, olive oil and nuts, reduces CVD and its risk factors [12,13]. Short-term RCTs have found little effect of fruits and vegetables on lipids and insulin resistance but perhaps small reductions in blood pressure [14–18].

The observed associations of fruit and vegetable consumption with health in Western settings may be due to beneficial effects of their nutrients and/or their replacement of less healthy foods. However, residual confounding by socioeconomic position (SEP) and other lifestyle factors [19] is also possible, because fruits and vegetables can be expensive. In western developed countries, fruit and vegetable consumption is associated with higher SEP [19]. Evidence from non-western settings with a potentially different confounding structure may help identify whether these associations are biologically based or contextually specific results of unmeasured confounding [20]. A recent prospective cohort study in Chinese found neither fruit nor vegetable consumption associated with ischemic heart disease (IHD) [21]. Moreover in previous observational studies, both in China and the West, the negative associations of fruit and vegetable consumption with CVD risk was attenuated by adjusting for health status [21,22], suggesting that the observed associations could partially be due to reverse causality. We examined the associations of fruit and vegetable consumption with CVD risk factors in a large study of older adults (50+ years) from the economically developing non-western setting of Guangzhou in Southern China, where vegetables are relatively cheap [23], but fruits are more expensive [23]. We also examined whether the associations varied by health status, and by sex and among men by smoking status.

## Materials and Methods

### Ethics statement

The Guangzhou Medical Ethics Committee of the Chinese Medical Association approved the study and all participants gave written, informed consent before participation.

### Participants

The Guangzhou Biobank Cohort Study (GBCS) is a collaboration among Guangzhou No. 12 Hospital and the Universities of Hong Kong and Birmingham and has been described in detail elsewhere [24]. Participants were recruited from “The Guangzhou Health and Happiness Association for the Respectable Elders”, a community social and welfare association unofficially aligned with the municipal government where membership is open to anyone aged 50+ years for a nominal monthly fee of 4 Yuan (50 US cents). Participants were recruited into the study in three recruitment phases: phase 1 from September 2003 to September 2004, phase 2 from April 2005 to May 2006, and phase 3 from September 2006 to January 2008. About 7% of permanent Guangzhou residents aged 50+ years are members of the association, of whom 33% enrolled and were included if they were capable of consenting, ambulatory, and not receiving treatment modalities which if omitted may result in immediate life-threatening risk, such as chemotherapy or radiotherapy for cancer, or dialysis for renal failure. Participants underwent a half-day detailed medical interview and examination, including lifestyle and other risk factors, disease history and physical examination.

In brief, we recorded seated blood pressure as the average of the last 2 of 3 measurements, using the Omron 705CP sphygmomanometer (Omron Corp, Kyoto, Japan). Standing height, without shoes, was measured to the nearest 0.1 cm. Weight (in light clothing) was measured to the nearest 0.1 kg. Body mass index (BMI) was calculated as weight (kg)/height (m)^2^. Hip circumference was measured to the nearest 0.1 cm at the greatest circumference around the buttocks below the iliac crest. Waist circumference was measured to the nearest 0.1 cm horizontally around the smallest circumference between the ribs and iliac crest, or at the level of the navel for obese participants. Low density lipoprotein (LDL)-cholesterol, high density lipoprotein (HDL)-cholesterol and fasting glucose were determined with a Shimadzu CL-8000 clinical chemical analyzer (Shimadzu Corp, Kyoto, Japan) in the hospital laboratory.

### Exposures

Fruits and vegetables were considered separately as 80 gram portions per day [25], obtained from a validated food frequency questionnaire (FFQ) [26] used in phases 1 and 2, but not in phase 3 [24]. This FFQ contains 300 food items, including commonly eaten fruits (29) and vegetables (47) from Southern China. The participants were asked the usual amount and frequency of each fruit and vegetable consumed in the past week, with a usual serving size specified. Vegetables were not separated into fresh, canned or frozen, although fruits were separated into fresh or dried, and were converted into equivalent amounts. Juice consumption is not common in this population (only 1% ever consumed juice in the past 7 days), so it was not included in the analysis. Beans were included as vegetables, nuts were not included.

### Outcomes

The primary outcome was the Framingham score [27], which predicts IHD risk from sex, age, LDL-cholesterol, HDL-cholesterol, blood pressure, and history of diabetes, with smoking removed to examine biological CVD risk. A higher score predicts higher IHD risk, consistent with the updated risk assessment equations for atherosclerotic CVD in the 2013 American College of Cardiology and the American Heart Association guidelines [28]. The Framingham score used to over-estimate IHD risk in Chinese populations, but ranked correctly [29]; it may now require recalibration to predict CVD risk correctly [30]. The secondary outcomes were its components.

### Health status

We constructed a 9-item index to assess health status, similar to one strongly associated with mortality [31], by counting chronic conditions (self-reported heart disease, stroke, diabetes, chronic obstructive pulmonary disease (COPD) and/or asthma, and hypertension), use of health services (regular use of medication and any hospital admission in the last 6 months), cognitive impairment (delayed recall score of 3 or less out of 10), and weight loss of more than 2.5 kg in the last 12 months.

### Statistical analysis

Multivariable linear regression was used to assess the adjusted associations of fruit and vegetable consumption with the Framingham score. Multivariable censored linear regression was used to assess associations with blood pressure, lipids and fasting glucose, because taking medications for hypertension (n = 4544), hyperlipidemia (n = 1339) or diabetes (n = 1587) might result in lower blood pressure, LDL-cholesterol and fasting glucose than the true measure, but higher HDL-cholesterol than the true measure. These models censored the outcomes for those on medication at the observed value so that the true measurement for blood pressure, LDL-cholesterol and fasting glucose was assumed to be that observed or higher, while for HDL-cholesterol it was assumed to be that observed or lower. Model 1 adjusted for sex, age (in 5-year age groups) and phase. Model 2 additionally adjusted for life course SEP (father’s occupation, education, longest-held occupation and household income per head) and lifestyle (smoking, alcohol use and physical activity). Model 3 additionally adjusted for health status. Model 4 additionally adjusted for BMI and waist-hip ratio (WHR), considering the unclear role of fruit and vegetable consumption in adiposity.

To assess reverse causality, we examined whether associations varied by health status, because people in poor health might consume more fruits and vegetables as a protective measure, which would generate different associations of fruit and vegetable consumption with CVD risk factors by health status. We assessed differences by health status from the p-value of the relevant interaction term in models including interactions with other confounders to avoid confounding by these interactions. We also similarly examined whether the associations varied by sex, and among men by smoking status.

Information on potential confounders was missing for less than 5%, apart from longest-held occupation (missing for 14.21%) and father’s occupation (missing for 42.62%). Missing potential confounders were predicted based on a flexible additive regression model with predictive mean matching incorporating exposures, potential confounders and outcomes. We imputed values 10 times and results from these imputations were summarized into single estimated beta-coefficients with confidence intervals and p-values adjusted for the missing data uncertainty [32]. A P-value<0.05 was considered as statistically significant.

### Sensitivity analysis

Given we previously found SEP was not clearly associated with CVD risk factors among men [33], we analyzed men separately, where socioeconomic confounding might be less relevant.

## Results

Of the original 20,305 participants in phases 1 (10,389) and 2 (9916) of GBCS, 19,518 had complete information on the outcomes. There were more women (13,916) than men (5602), and the women were younger (mean age 62.0 (standard deviation (SD) 6.7) than the men (mean age 64.7 (SD 6.3)). Fruit consumption ranged from 0.0 to 12.4 portions/day (80 gram/portion), with a median of 1.5 portions/day (mean 1.7 (SD 1.4)), 95% of participants ate less than 4.3 portions/day. Vegetable consumption ranged from 0.0 to 12.5 portions/day, with a median of 2.3 portions/day (mean 2.8 SD (1.7)), 95% of participants ate less than 6.3 portions/day. Both vegetable and fruit consumption were lower than the recommendations [2].

Table 1 shows fruit consumption was associated with higher education, non-manual occupation of father, a non-manual job, higher household income, less smoking, less use of alcohol, more physical activity in men and women, higher BMI and WHR in men, higher BMI, lower WHR and good health status in women. Vegetable consumption was associated with non-manual occupation of father, a non-manual job, higher household income, less use of alcohol, more physical activity, higher BMI, higher WHR and poor health status in men and women.

**Table 1:** Characteristics by Fruit and Vegetable Consumption in Older Chinese (5602 men and 13,916 women) in Phases 1 and 2 of the Guangzhou Biobank Cohort Study, 2003–2006.

			Fruit consumption				Vegetable consumption			
	Gender		(80 gram portion/day)				(80 gram portion/ day)			
	Men	Women	Men	^a^ *P*-value	Women	^a^ *P*-value	Men	^a^ *P*-value	Women	^a^ *P*-value
N	5602	13916								
Age group (%)										
50–54	6.4	16.4	1.70		1.85		2.34		2.29	
55–59	18.3	27.6	1.70		1.83		2.89		2.86	
60–64	25.5	21.0	1.79		1.74		3.06		2.94	
65–69	27.5	21.5	1.75		1.68		3.05		2.83	
70–74	18.8	11.3	1.79		1.50		2.91		2.53	
75–79	3.0	1.9	1.81		1.53		3.04		2.79	
≥80	0.5	0.3	1.86	0.10	1.85	**<0.001**	2.75	**0.001**	3.13	**<0.001**
Education (%)										
Less than primary school	2.7	14.0	1.40		1.34		3.08		2.68	
Primary school	28.0	37.6	1.55		1.64		2.91		2.76	
Junior middle school	28.7	24.7	1.74		1.84		2.93		2.74	
Senior middle school	22.8	18.0	1.91		2.01		2.96		2.71	
Junior college	9.4	3.7	1.93		2.13		2.84		2.72	
College or above	8.4	2.0	1.98	**<0.001**	2.10	**<0.001**	3.20	0.14	3.13	0.11
Father’s occupation (%)										
Manual	79.5	78.8	1.71		1.68		2.83		2.67	
Non-manual	20.5	21.2	1.83	**0.04**	1.86	**<0.001**	3.07	**0.002**	2.76	**0.04**
Job type (%)										
Manual	54.1	77.3	1.64		1.63		2.88		2.74	
Non-manual	46.0	22.7	1.89	**<0.001**	2.01	**<0.001**	3.19	**<0.001**	3.09	**<0.001**
Income group (%)										
< 10000 yuan	22.2	45.0	1.60		1.62		2.80		2.76	
10000 to 15000 yuan	45.2	43.6	1.73		1.82		2.94		2.66	
≥ 15000 yuan	32.6	11.4	1.92	**<0.001**	1.95	**<0.001**	3.03	**0.003**	2.80	**<0.001**
Smoking status (%)										
Never	41.1	95.9	1.94		1.75		3.02		2.74	
Ex-smoker	29.3	1.9	1.84		1.58		2.97		2.93	
Current smoker	29.6	2.3	1.43	**<0.001**	1.41	**<0.001**	2.83	**0.01**	2.58	**0.048**
Alcohol use (%)										
Never	60.7	89.6	1.73		1.70		2.88		2.71	
<1/month	14.5	6.3	1.93		2.21		3.05		3.02	
<1/week	4.4	1.1	1.72		2.01		2.96		3.19	
1-4/week	8.4	1.1	1.66		1.79		2.85		3.06	
>5/week	9.4	1.3	1.67		1.69		3.31		2.97	
Ex-drinker	2.7	0.6	1.73	**0.004**	1.88	**<0.001**	3.50	**<0.001**	3.88	**<0.001**
Physical activity (IPAQ) (%)										
Inactive	8.6	8.1	1.43		1.43		2.00		1.87	
Minimally active	49.3	46.7	1.69		1.69		2.73		2.49	
HEPA	42.2	45.2	1.90	**<0.001**	1.85	**<0.001**	3.40	**<0.001**	3.15	**<0.001**
BMI	23.5	23.9	0.03	**<0.001**	0.02	**<0.001**	0.04	**<0.001**	0.03	**<0.001**
WHR	0.90	0.86	0.73	**0.02**	-0.86	**<0.001**	1.31	**0.001**	0.79	**0.001**
Health status										
Good health	32.6	34.2	0.69		0.73		1.13		1.06	
Poor health	67.4	65.8	0.71	0.25	0.68	**<0.001**	1.21	**<0.001**	1.11	**<0.001**

Abbreviations: IPAQ, International Physical Activity Questionnaire; HEPA, health-enhancing physical activity, i.e., vigorous activity at least 3 days a week that corresponds to a minimum of 1500 metabolic equivalent (MET) minutes per week, or activity 7 days of the week that corresponds to at least 3000 MET minutes per week.

^a^ *P* value from chi-square test for categorical variables and from one-way analysis of variants (ANOVA) for continuous variables, 2 sided; bold values indicate *P*<0.05.

Table 2 shows fruit consumption was not associated with the Framingham score. It was associated with higher diastolic blood pressure, lower HDL-cholesterol and lower fasting glucose, but not with systolic blood pressure or LDL-cholesterol. The associations varied by health status for the Framingham score and fasting glucose. Greater fruit consumption was associated with a lower Framingham score and with lower fasting glucose in participants in poor health, but with a higher Framingham score and higher fasting glucose in participants in good health. The associations did not vary by sex, except greater fruit consumption was associated with higher diastolic blood pressure only among women.

**Table 2:** Adjusted Associations of Fruit Consumption with Framingham Risk Score and CVD Risk Factors after Multiple Imputation in Older Chinese in Phases 1 and 2 of the Guangzhou Biobank Cohort Study, 2003–2006.

	Fruit consumption				*P*-value for	*P*-value for
	(80 gram portion/day)				interaction	interaction by
	^b^Model	N	^c^Coefficient	95%CI	by sex	health status
^a^Framingham	1	19518	**-0.04**	-0.07, -0.01		
Risk Score	2	19518	-0.02	-0.05, 0.01	0.87	**<0.001**
	3	19518	-0.01	-0.04, 0.02		
	4	19518	**-0.03**	-0.06, -0.004		
	1	6588	**0.05**	0.01, 0.08		
Good health	2	6588	**0.05**	0.02, 0.09		
	4	6588	**0.04**	0.003, 0.07		
	1	12930	**-0.06**	-0.09, -0.02		
Poor health	2	12930	**-0.04**	-0.08, -0.004		
	4	12930	**-0.06**	-0.10, -0.03		
Systolic blood	1	19441	-0.07	-0.34, 0.20		
pressure	2	19441	0.04	-0.23, 0.32	0.22	0.99
(mm Hg)	3	19441	0.18	-0.05, 0.42		
	4	19441	-0.04	-0.27, 0.19		
Diastolic blood	1	19440	**0.17**	0.04, 0.31		
pressure	2	19440	**0.21**	0.07, 0.35	**0.02**	0.41
(mm Hg)	3	19440	**0.28**	0.15, 0.40		
	4	19440	**0.14**	0.01, 0.26		
	1	5574	0.10	-0.15, 0.35		
Men	2	5574	0.004	-0.25, 0.26		
	3	5574	0.04	-0.19, 0.28		
	4	5574	-0.12	-0.35, 0.10		
	1	13866	**0.22**	0.05, 0.38		
Women	2	13866	**0.32**	0.15, 0.48		
	3	13866	**0.39**	0.23, 0.54		
	4	13866	**0.26**	0.11, 0.41		
HDL-cholesterol	1	19481	**-0.01**	-0.01, -0.002		
(mmol/L)	2	19481	**-0.01**	-0.01, -0.005	0.16	0.45
	3	19481	**-0.01**	-0.01, -0.005		
	4	19481	**-0.01**	-0.01, -0.003		
LDL-cholesterol	1	19481	**0.01**	0.002, 0.02		
(mmol/L)	2	19481	0.01	-0.001, 0.01	0.06	0.47
	3	19481	0.01	-0.001, 0.01		
	4	19481	0.004	-0.003, 0.01		
Fasting glucose	1	19421	**-0.05**	-0.07, -0.03		
(mmol/L)	2	19421	**-0.05**	-0.07, -0.03	0.68	**<0.001**
	3	19421	**-0.05**	-0.07, -0.03		
	4	19421	**-0.05**	-0.07, -0.03		
	1	6561	**0.02**	0.01, 0.03		
Good health	2	6561	**0.02**	0.01, 0.03		
	4	6561	**0.01**	0.004, 0.02		
	1	12860	**-0.09**	-0.12, -0.06		
Poor health	2	12860	**-0.09**	-0.12, -0.06		
	4	12860	**-0.09**	-0.12, -0.06		

^a^Multivariable linear regression was used for Framingham risk score; multivariable censored linear regression was used for blood pressure, cholesterol and glucose.

^b^Model 1 adjusted for age, sex and phase; Model 2 additionally adjusted for SEP (education, father’s occupation, longest-held occupation and personal income) and lifestyle (smoking status, alcohol use and physical activity); Model 3 additionally adjusted for baseline health status; Model 4 additionally adjusted for BMI and WHR.

^c^Coefficient means changes in risk score; bold values indicate *P*<0.05.

Table 3 shows greater vegetable consumption was associated with a higher Framingham score, higher blood pressure and higher fasting glucose, but not with HDL-cholesterol or LDL-cholesterol. The associations did not vary by health status, or sex except that greater vegetable consumption was associated with higher fasting glucose only among men.

**Table 3:** Adjusted Associations of Vegetable Consumption with Framingham Risk Score and CVD Risk Factors after Multiple Imputation in Older Chinese in Phases 1 and 2 of the Guangzhou Biobank Cohort Study, 2003–2006.

	Vegetable consumption				*P*-value for	*P*-value for
	(80 gram portion/day)				interaction	interaction b
	^b^Model	N	^c^Coefficient	95%CI	by sex	health status
^a^Framingham	1	19518	**0.05**	0.02, 0.07		
Risk Score	2	19518	**0.06**	0.03, 0.08	0.97	0.51
	3	19518	**0.03**	0.01, 0.05		
	4	19518	0.02	-0.002, 0.04		
Systolic blood	1	19441	**0.49**	0.27, 0.72		
pressure	2	19441	**0.56**	0.34, 0.79	0.98	0.88
(mm Hg)	3	19441	**0.30**	0.10, 0.50		
	4	19441	0.17	-0.03, 0.37		
Diastolic blood	1	19440	**0.27**	0.16, 0.39		
pressure	2	19440	**0.30**	0.18, 0.42	0.85	0.14
(mm Hg)	3	19440	**0.18**	0.07, 0.29		
	4	19440	0.10	-0.01, 0.20		
HDL-cholesterol	1	19481	-0.001	-0.004, 0.003		
(mmol/L)	2	19481	-0.002	-0.01, 0.002	0.15	0.29
	3	19481	-0.002	-0.01, 0.002		
	4	19481	-0.001	-0.004, 0.003		
LDL-cholesterol	1	19481	-0.002	-0.01, 0.003		
(mmol/L)	2	19481	-0.003	-0.01, 0.003	0.50	0.97
	3	19481	-0.004	-0.01, 0.002		
	4	19481	-0.01	-0.01, 0.003		
Fasting glucose	1	19421	**0.03**	0.02, 0.05		
(mmol/L)	2	19421	**0.03**	0.02, 0.05	**0.01**	0.08
	3	19421	**0.03**	0.01, 0.04		
	4	19421	**0.02**	0.01, 0.04		
	1	5570	**0.06**	0.04, 0.09		
Men	2	5570	**0.07**	0.04, 0.09		
	3	5570	**0.06**	0.03, 0.08		
	4	5570	**0.05**	0.03, 0.08		
	1	13851	0.02	-0.004, 0.04		
Women	2	13851	0.02	-0.002, 0.04		
	3	13851	0.01	-0.01, 0.03		
	4	13851	0.01	-0.01, 0.03		

^a^Multivariable linear regression was used for Framingham risk score; multivariable censored linear regression was used for blood pressure, cholesterol and glucose.

^b^Model 1 adjusted for age, sex and phase; Model 2 additionally adjusted for SEP (education, father’s occupation, longest-held occupation and personal income) and lifestyle (smoking status, alcohol use and physical activity); Model 3 additionally adjusted for baseline health status; Model 4 additionally adjusted for BMI and WHR.

^c^Coefficient means changes in risk score; bold values indicate *P*<0.05.

Table 4 shows that in men fruit consumption was not associated with the Framingham score, blood pressure or LDL-cholesterol, but with lower HDL-cholesterol and lower fasting glucose. Most associations did not vary by health status, or smoking status, except that greater fruit consumption was only associated with lower fasting glucose in men in poor health.

**Table 4:** Adjusted Associations of Fruit Consumption with Framingham Risk Score and CVD Risk Factors after Multiple Imputation in Older Chinese Men in Phases 1 and 2 of the Guangzhou Biobank Cohort Study, 2003–2006.

	Fruit consumption				*P*-value for	*P*-value for
	(80 gram portion/day)				interaction by	interaction
	^b^Model	N	^c^Coefficient	95%CI	health status	by smoking
^a^Framingham	1	5602	0.01	-0.03, 0.04		
Risk Score	2	5602	-0.004	-0.04, 0.03	0.47	0.94
	3	5602	-0.001	-0.04, 0.03		
	4	5602	-0.02	-0.05, 0.01		
Systolic blood	1	5575	0.07	-0.40, 0.54		
pressure	2	5575	-0.10	-0.58, 0.38	0.78	0.12
(mm Hg)	3	5575	-0.02	-0.44, 0.41		
	4	5575	-0.29	-0.70, 0.11		
Diastolic blood	1	5574	0.10	-0.16, 0.35		
pressure	2	5574	-0.001	-0.26, 0.26	0.89	0.06
(mm Hg)	3	5574	0.03	-0.20, 0.27		
	4	5574	-0.14	-0.36, 0.09		
HDL-cholesterol	1	5591	**-0.01**	-0.02, -0.01		
(mmol/L)	2	5591	**-0.01**	-0.02, -0.01	0.98	0.38
	3	5591	**-0.01**	-0.02, -0.01		
	4	5591	**-0.01**	-0.02, -0.004		
LDL-cholesterol	1	5591	-0.003	-0.01, 0.01		
(mmol/L)	2	5591	-0.004	-0.02, 0.01	0.61	0.87
	3	5591	-0.004	-0.02, 0.01		
	4	5591	-0.01	-0.02, 0.005		
Fasting glucose	1	5570	-0.03	-0.06, 0.002		
(mmol/L)	2	5570	**-0.04**	-0.07, -0.01	**0.02**	0.38
	3	5570	**-0.04**	-0.07, -0.01		
	4	5570	**-0.05**	-0.08, -0.02		
	1	1822	**0.03**	0.01, 0.05		
Good health	2	1822	0.02	-0.004, 0.03		
	4	1822	0.01	-0.01, 0.03		
	1	3748	**-0.06**	-0.11, -0.02		
Poor health	2	3748	**-0.07**	-0.12, -0.03		
	4	3748	**-0.07**	-0.12, -0.03		

^a^Multivariable linear regression was used for Framingham risk score; multivariable censored linear regression was used for blood pressure, cholesterol and glucose.

^b^Model 1 adjusted for age, and phase; Model 2 additionally adjusted for SEP (education, father’s occupation, longest-held occupation and personal income) and lifestyle (smoking status, alcohol use and physical activity); Model 3 additionally adjusted for baseline health status; Model 4 additionally adjusted for BMI and WHR.

^c^Coefficient means changes in risk score; bold values indicate *P*<0.05.

Table 5 shows that in men greater vegetable consumption was associated with a higher Framingham score, higher blood pressure and higher fasting glucose, but not with HDL-cholesterol or LDL-cholesterol. Most associations did not vary by health status, or smoking status, except that greater vegetable consumption was associated with higher fasting glucose in men in poor health.

**Table 5:** Adjusted Associations of Vegetable Consumption with Framingham Risk Score and CVD Risk Factors after Multiple Imputation in Older Chinese Men in Phases 1 and 2 of the Guangzhou Biobank Cohort Study, 2003–2006.

	Vegetable consumption				P-value for	P-value for
	(80 gram portion/day)				interaction by	interaction
	^b^Model	N	^c^Coefficient	95%CI	health status	by smoking
^a^Framingham	1	5602	**0.06**	0.03, 0.09		
Risk Score	2	5602	**0.06**	0.03, 0.09	0.96	0.71
	3	5602	**0.04**	0.01, 0.06		
	4	5602	0.02	-0.01, 0.05		
Systolic blood	1	5575	**0.69**	0.30, 1.08		
pressure	2	5575	**0.65**	0.26, 1.04	0.65	0.15
(mm Hg)	3	5575	0.30	-0.04, 0.65		
	4	5575	0.13	-0.21, 0.46		
Diastolic blood	1	5574	**0.35**	0.14, 0.56		
pressure	2	5574	**0.33**	0.12, 0.54	0.86	0.34
(mm Hg)	3	5574	0.17	-0.03, 0.36		
	4	5574	0.06	-0.13, 0.24		
	1	5591	-0.01	-0.01, 0.001		
HDL-cholesterol	2	5591	-0.01	-0.01, 0.0001	0.29	0.30
	3	5591	-0.01	-0.01, 0.0003		
(mmol/L)	4	5591	-0.004	-0.01, 0.002		
	1	5591	-0.01	-0.02, 0.004		
LDL-cholesterol	2	5591	-0.01	-0.02, 0.004	0.36	0.84
	3	5591	-0.01	-0.02, 0.002		
(mmol/L)	4	5591	**-0.01**	-0.02, -0.0002		
Fasting glucose	1	5570	**0.06**	0.04, 0.09		
(mmol/L)	2	5570	**0.07**	0.04, 0.09	**0.01**	0.61
	3	5570	**0.06**	0.03, 0.08		
	4	5570	**0.05**	0.03, 0.08		
	1	1826	0.01	-0.01, 0.03		
Good health	2	1826	0.004	-0.01, 0.02		
	4	1826	0.001	-0.02, 0.02		
	1	3744	**0.08**	0.04, 0.11		
Poor health	2	3744	**0.08**	0.04, 0.12		
	4	3744	**0.07**	0.04, 0.11		

^a^Multivariable linear regression was used for Framingham risk score; multivariable censored linear regression was used for blood pressure, cholesterol and glucose.

^b^Model 1 adjusted for age, and phase; Model 2 additionally adjusted for SEP (education, father’s occupation, longest-held occupation and personal income) and lifestyle (smoking status, alcohol use and physical activity); Model 3 additionally adjusted for baseline health status; Model 4 additionally adjusted for BMI and WHR.

^c^Coefficient means changes in risk score; bold values indicate *P*<0.05.

## Discussion

This large study from an under-studied non-western developing population showed no clear association of fruit consumption with the Framingham score. Greater fruit consumption was associated with slightly lower HDL-cholesterol and slightly lower fasting glucose. Greater vegetable consumption was associated with a slightly higher Framingham score, blood pressure and fasting glucose. In men, where SEP has little association with CVD risk factors in this setting [33], these same associations were also evident. Negative associations of fruit and vegetable consumption with cardiovascular risk factors were more evident for those in poor health, whilst positive associations were more evident for those in good health, suggesting these might be the result of changes in response to poor health.

Our observations that fruit consumption was not associated with the Framingham score and that vegetable consumption was associated with a higher Framingham score are inconsistent with most studies from Western countries, where fruit and vegetable consumption is usually associated with lower CVD and its risk factors [3,4,12,13]. Moreover, in our setting where among men less confounding by SEP might exist [33], fruit and vegetable consumption was not clearly associated with lower values of IHD risk factors. As such, our findings are consistent with a recent prospective cohort study in Chinese men, where neither fruit nor vegetable consumption was associated with IHD [21]. The differences observed in the associations for fruits and vegetables could be due to different nutrient composition, different patterns of residual confounding, or different processing methods, for sodium may be added to vegetables but not to fruit.

Different observed associations by setting might be due to differences in the fruits and vegetables commonly consumed or differences in cooking methods. Nutrients such as vitamins and some phenolic compounds may be lost due to Southern Chinese cooking methods (stir-frying, steaming, boiling and roasting), or fiber may be lost during processing [34]. However, most vitamins do not protect against CVD [11]. Salty sauces used in China [35], might counteract the benefits of vegetables [36], because dietary sodium could raise blood pressure [14,37,38]. Alternatively, these differences by setting could be due to confounding by SEP in Western settings, given both higher fruit and vegetable consumption and lower IHD risk factors are associated with higher SEP. Differences could also be due to reverse causality, assuming Chinese people are more willing to change their diet in response to ill-health than Westerners, perhaps as part of a more holistic attitude to medicine. However, the positive associations of vegetable consumption with the Framingham score were evident for participants in good health and poor health, making reverse causation unlikely. Finally, these different observations by setting could be arising because fruit and vegetables are correlated with other dietary items that are hypothesized to protect against CVD such as less processed red meat consumption [39], or correlated with other lifestyle factor such as less smoking and more physical activity [19,40], but the correlations vary by setting, such that fruit and vegetables are more strongly correlated with protective dietary items or healthier lifestyle factors in Western settings but less so in this setting.

Our results are more consistent with RCTs where fruits and vegetables have little or no effect on blood pressure, lipids or insulin resistance [14–18]. Most vitamins also have little beneficial effect, whereas sugar may be harmful [11,41]. A meta-analysis of RCTs found fructose from fruit increased total cholesterol and LDL-cholesterol, and did not reduce HDL-cholesterol [42]. Overall, our results are more consistent with the emerging view that adult diet actually has fairly small effects on health, which could easily be inflated by biases in observational studies such as the confounding by SEP [43]. A greater understanding of the mechanisms by which diet affects CVD risk might help clarify the role of diet in CVD, both for fruit and vegetables and more broadly.

Strengths of our study include a large sample from an understudied population, whose lifetime experiences are typical of much of the global population. Nevertheless, limitations existed. First, participants were not a randomly selected, representative sample. However, sample selection should not affect internal associations, unless we missed people with specific combinations of fruit and vegetable consumption and CVD risk factors. Second, we used recalled consumption of fruits and vegetables. Any non-differential misclassification would underestimate the true effects. Systematic recall bias by CVD risk factors is unlikely as participants were unaware of this hypothesis at the time of interview. Third, we did not examine the role of each specific fruit and vegetable, so some specific fruits and vegetables may be beneficial for health. Fourth, we did not examine the association of fruits and vegetables with CVD events, because the follow-up to collect and verify CVD events has not yet been completed. However, the components of the Framingham score, such as blood pressure, lipids and diabetes, are important intervention targets in their own right. Fourth, the causes of ischemic and hemorrhage diseases might be different, for example, total and HDL-cholesterol have opposite associations with the risk of hemorrhage stroke and cerebral infarction [44]. Thus it is possible that the association of diet with IHD or atherosclerotic CVD may be different from that with hemorrhage stroke. Finally, although our study suggests observations concerning the associations of fruit and vegetable consumption with CVD risk factors may be contextually specific to Western populations, our findings should be interpreted cautiously as with all observational studies, particularly because of their cross-sectional nature, although they are fairly consistent with meta-analyses of RCTs [10,42]. Besides, our results that fruit or vegetable consumption was associated with negligible differences in the Framingham scores, does not necessarily imply that fruit and vegetables have little association with overall health benefits or specifically with cardiovascular risk. The Framingham score includes CVD risk factors, such as blood pressure, LDL-cholesterol, HDL-cholesterol and fasting glucose. Fruit and vegetables may have effects on CVD independent of these risk factors.

## Conclusion

This large study from a non-western setting found little association of fruit or vegetables with the Framingham score, or major CVD risk factors. It might indicate that observations concerning the benefits of fruit and vegetable consumption may be contextually specific to the West, or that the health benefits of fruit and vegetables operate via other mechanisms than these risk factors whose elucidation could provide new means of preventing or treating a leading cause of death globally.

## Supporting Information

S1 File The associations of fruit and vegetable consumption with Framingham score and its components. Fruit and vegetable consumption with the Framingham score (Figure A). Fruit and vegetable consumption with systolic blood pressure (Figure B). Fruit and vegetable consumption with diastolic blood pressure (Fgure C). Fruit and vegetable consumption with HDL-cholesterol (Figure D). Fruit and vegetable consumption with LDL-cholesterol (Figure E). Fruit and vegetable consumption with plasma glucose (Figure F).
(DOCX)
