# Supplementary material for: Fruit and Vegetable Consumption and Cardiovascular Risk Factors in Older Chinese: The Guangzhou Biobank Cohort Study
Source: PLoS One. 2015 Aug 10;10(8):e0135380. doi: 10.1371/journal.pone.0135380 (PMC4530892; doi:10.1371/journal.pone.0135380)

S1 Figure A Fruit and vegetable consumption with the Framingham score


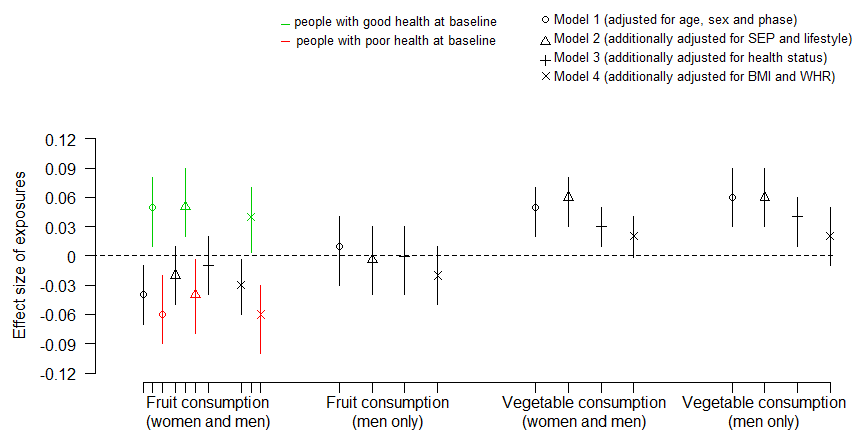


S2
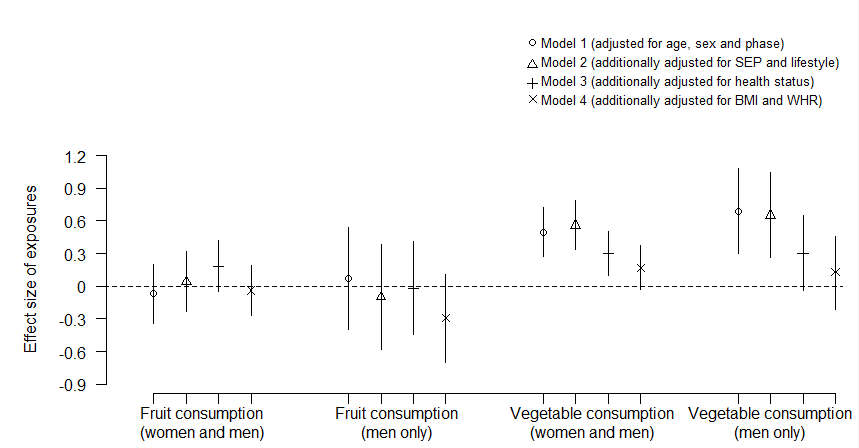
Figure B Fruit and vegetable consumption with systolic blood pressure

S3 Figure C Fruit and vegetable consumption with diastolic blood pressure
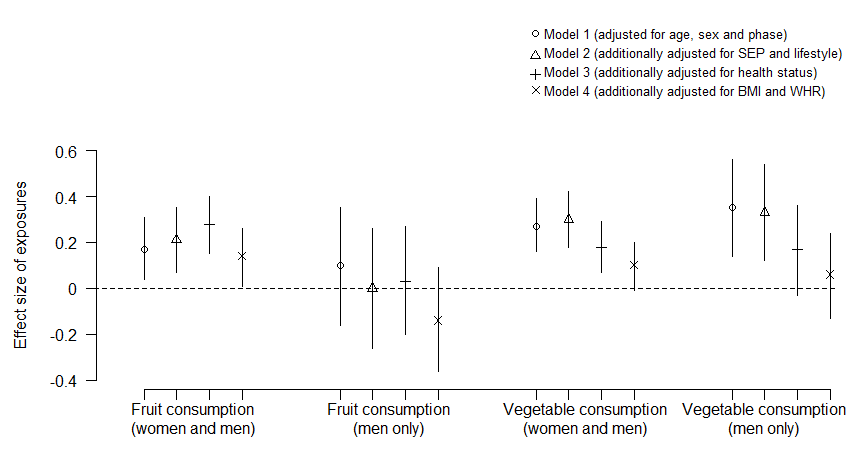


S4 Figure D Fruit and vegetable consumption with HDL-cholesterol
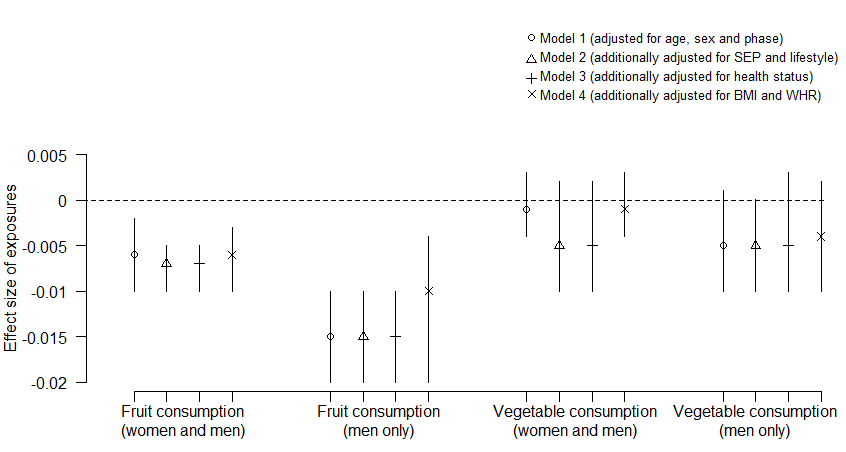


S5 Figure E Fruit and vegetable consumption with LDL-cholesterol


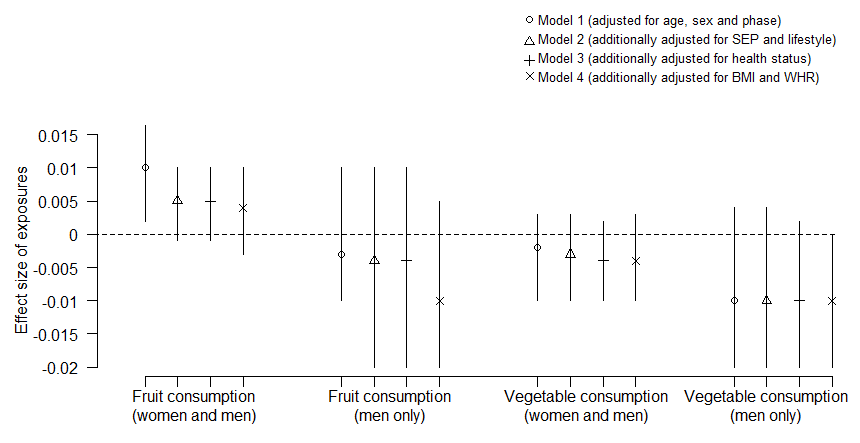


S6 Figure F Fruit and vegetable consumption with plasma glucose


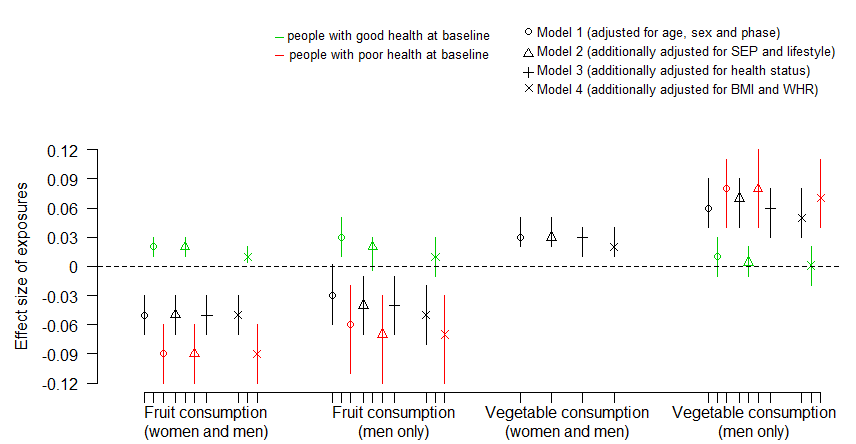

Supplement: S1 File — Fruit and vegetable consumption with the Framingham score (Figure A). Fruit and vegetable consumption with systolic blood pressure (Figure B). Fruit and vegetable consumption with diastolic blood pressure (Fgure C). Fruit and vegetable consumption with HDL-cholesterol (Figure D). Fruit and vegetable consumption with LDL-cholesterol (Figure E). Fruit and vegetable consumption with plasma glucose (Figure F). (DOCX) [file pone.0135380.s001.docx]
